# Synthesis, Characterization and Biocompatibility Evaluation of Novel Chitosan Lipid Micro-Systems for Modified Release of Diclofenac Sodium

**DOI:** 10.3390/biomedicines11020453

**Published:** 2023-02-04

**Authors:** Ana-Maria Raluca Pauna, Liliana Mititelu Tartau, Maria Bogdan, Andreea-Daniela Meca, Gratiela Eliza Popa, Ana Maria Pelin, Cristian Ilie Drochioi, Daniela Angelica Pricop, Liliana Lacramioara Pavel

**Affiliations:** 1 Department of Pharmacology, Faculty of Medicine, “Grigore T. Popa” University of Medicine and Pharmacy, 700115 Iasi, Romania; 2 Department of Pharmacology, Faculty of Pharmacy, University of Medicine and Pharmacy, 200349 Craiova, Romania; 3 Department of Pharmaceutical Technology, Faculty of Pharmacy, “Grigore T. Popa” University of Medicine and Pharmacy, 700115 Iasi, Romania; 4 Department of Pharmaceutical Sciences, Faculty of Medicine and Pharmacy, “Dunărea de Jos” University, 800010 Galați, Romania; 5 Surgical Department, Faculty of Dental Medicine, University of Medicine and Pharmacy, 700115 Iasi, Romania; 6 Department of Physics, Faculty of Physics, “Al. I. Cuza” University, 700506 Iasi, Romania; 7 Department of Morphological and Functional Sciences, Faculty of Medicine and Pharmacy, “Dunărea de Jos” University, 800010 Galați, Romania

**Keywords:** chitosan, diclofenac, lipid vesicles, rats, characterization, biocompatibility

## Abstract

The purpose of our study was the obtaining, characterization and biocompatibility estimation of novel carrier systems for diclofenac. Diclofenac is a potent nonsteroidal anti-inflammatory drug with frequent gastrointestinal side effects, impairing the quality of the patient’s life. Original diclofenac-loaded micro-vesicles coated with chitosan were prepared and physico-chemical analyzed. We investigated their in vitro hemocompatibility and in vivo biocompatibility in rats. The animals were treated orally as follows: group 1 (Control): distilled water 0.3 mL/100 g body weight; Group 2 (CHIT): 0.3 mL/100 g body weight 0.5% chitosan solution; Group 3 (DCF): 15 mg/kg body weight diclofenac; Group 4 (DCF-ves): lipid vesicles loaded with diclofenac 15 mg/kg body weight. Blood samples were collected for assessing: red blood cells, hemoglobin, hematocrit and leukocyte formula. A series of specific parameters of the liver and kidney function, some markers of immune defense, as well as the activity of some enzymes involved in oxidative processes, were also investigated. At the end of the experiment, the animals were sacrificed and fragments of liver, kidney and stomach were collected for histopathological examination. No blood hemolysis was evidenced by the in vitro test with the administration of diclofenac vesicles. The animals treated with diclofenac lipid vesicles stabilized with chitosan did not display any notable differences in their hematological and biochemical profile compared to control animals. These data correlated with the histological results, which showed the absence of architectural changes in the examined tissues. Biological in vitro and in vivo evaluation revealed that the microvesicles containing diclofenac are biocompatible, with potential to be used as delivery systems to modify the drug release, thus making them an attractive candidate for biomedical applications.

## 1. Introduction

Significant progress has been made in the field of nanotechnology, especially in medicine, in the last years [1,2,3,4,5,6]. The medical application of nanotechnologies, usually called nanomedicine, has given a decisive impetus to the development of various types of drug-carrying nano-systems, ranging in size from 1 to 1000 nm [7]. Multiple varieties of nano-transporters composed of different materials, such as lipids, polymers and inorganic substances, have been proposed for the biomedical field [8]. The obtained delivery systems are suitable to further use for different applications, depending on their physical and chemical properties [9,10]. 

In drug delivery technologies, nano-transporters are designed to: (i) protect a drug against in vivo degradation; (ii) increase the topical absorption of active substances by facilitating their diffusion to the epithelium; (iii) modify the distribution profile or other stages of drug pharmacokinetics in body tissues; and (iv) facilitate intracellular penetration and subcellular distribution [11]. In particular, the oral route is preferable because it is safe and easy-to-use with low costs, and the procedures for the preparation of nanoparticles do not require aseptic conditions [12]. The oral administration of nanoparticles incorporating drugs has the following advantages: optimization of solubility, increase in bioavailability, targeted action and controlled release of the active substance [13,14,15]. The use of chitosan in the design of nanoparticles, due to the covalent bonds it forms, ensures the stability of the systems against rapid degradation at the gastrointestinal level [13].

Moreover, the surface modification of pharmaceutical nano-transporters is normally used to enhance their pharmacodynamics effects. The most important results of such changes include: higher stability, prolonged half-life of circulating nano-transporters, passive or active targeting of the affected pathological area as well as increased reactivity to local stimuli, such as changes in pH and/or local temperature [16].

Carrier systems represented by biocompatible and biodegradable lipid vesicles present a series of limitations due to the risk of rapid elimination of the drug they contain, after administration in vivo. Therefore, different processes have been developed to modify the surface by coating the vesicles with different polymers. This led to improved stability, extending their life in the bloodstream and ensuring sustained release of the entrapped drug [12,13].One of the most challenging research directions is represented by the design and study of the approaches of incorporating the nonsteroidal anti-inflammatory drugs (NSAIDs), agents, commonly prescribed in medical practice in various pathological conditions accompanied by pain, fever or inflammation. NSAIDs are classified in various classes, with differences in their pharmacokinetic and pharmaco-toxicological profiles; thus, obtaining novel compounds by incorporating NSAIDs in nanoparticles is of great interest. Various experimental studies have shown that the use of NSAIDs nanoparticles has the advantage of significantly reducing renal and gastrointestinal side effects, usually produced by the unincorporated drug [17,18].

Although there are various reports in literature regarding the incorporation of NSAIDs into nanoparticles, there is relatively little information regarding their pharmacodynamics effects on inflammation patterns [19]. These systems have been characterized and have demonstrated effective and prolonged in vitro release [20,21,22], but data on their in vivo effects are inconsistent [23]. Moreover, it is possible that certain formulation parameters and polymers used in the design of these drug delivery systems may have a number of pharmacodynamics advantages, especially in terms of reducing the adverse effects of the active substance [19,24,25].

In the current study, we chose diclofenac (DCF) from the NSAID group, known to have strong anti-inflammatory effects, but also many side effects, especially in the gastrointestinal tract [26,27,28]. DCF is a phenylacetic acid derivative with an acidity constant of 4 (therefore considered a weak acid) and a partition coefficient of 13.4 (which indicates a partial solubility in both aqueous and hydrophobic media). The structural features of the molecule, namely the presence of the phenylacetic acid group and a phenyl ring containing two chlorine atoms, facilitate the maximum torsion of the phenyl ring [29], which ensures a good fit in the coupling to the appropriate substrate binding pocket at cyclooxygenase enzyme (COX) [30].

There is evidence that DCF inhibits lipoxygenases and activates the antinociceptive pathway of cyclic nitric oxide-guanosine monophosphate (GMP). It is also speculated that it may inhibit phospholipase A2 [31]. These additional actions may explain the high efficiency of DCF, which is considered one of the most potent NSAIDs [32].

In this paper, we consider that chitosan, being a biomedical material of natural origin, can fulfill the role of a polymer capable of modifying the surface properties of lipid vesicles, leading to an increase in their stability in suspension and their half-life in vivo. On the other hand, the adhesion to the cell membrane of chitosan could allow the efficiency of the controlled drug release process. In this study, we obtained and characterized innovative systems containing DCF, in order to highlight the fact that they present fewer adverse effects compared to the unincorporated drug. We aimed to characterize the original obtained systems from both physical and chemical point of view and to evaluate the in vivo biocompatibility after administration in laboratory animals.

## 2. Materials and Methods

### 2.1. Substances

The tested substance (DCF sodium) and those required for the preparation of micro-particles (chitosan, phosphatidylcholine, cholesterol, chloroform) were purchased from Sigma-Aldrich Chemical Co., Steinheim, Germany.

The utilized chitosan had a degree of N-deacetylation of 79.7%, an average molecular weight of Mw = 310,000 g/mol and a polydispersity index of 3.26. A solution of 0.5% (*w/w*) chitosan was prepared in a 0.5% (*v/v*) acetic acid solution. The lipid used was L-α-phosphatidylcholine, type XVI-E, approximately 99% pure. Bi-distilled water was purchased from Zentiva Company, Prague, Czechia.

### 2.2. Laboratory Animals

For the proposed laboratory investigations, 24 white healthy, non-genetically modified Wistar rats (3 months old), weighing 200–250 g, with a uniform distribution by sex (half males, half females) were used (University of Medicine and Pharmacy “Grigore T. Popa”, Iasi, Romania-Ethics Certificate No. 24/21.12.2020). The animals were purchased from the National Medical-Military Institute “Cantacuzino” for Research and Development, Baneasa Resort, Bucharest, Romania and brought to the biobase of the University of Medicine and Pharmacy “Grigore T. Popa”, Iasi within the CEMEX laboratory (“Advanced Center for Research and Development in Experimental Medicine–CEMEX”).

The shelter, the care and the investigations took place in a specially designed space within the biobase of the “Grigore T. Popa” University of Medicine and Pharmacy, Iasi, Romania and in the experimental research laboratory within CEMEX.

After 2 weeks of quarantine, the animals were brought to the test room the day before the experiment for accommodation purposes, being kept in standard laboratory conditions (constant temperature 21 °C ± 2 °C, relative humidity 50–70% and alternating lighting cycle (light/dark ratio = 12/12 h)).

The animals were fed with fully standardized solid food (pellets) adapted to the species. The amount of food ingested by each animal was monitored daily. Drinking water was administered *ad libitum* with the help of special devices, but during the experiments, the animals were deprived of food and fluids. In order to prevent chronobiological influences, experimental tests were performed between 8:00 a.m. and 12:00 each day.

The experiments were conducted in compliance with the principles of the 3Rs: replacement, reduction (reduce the suffering of the animal) and refinement (refine the method to reduce the pain of the animals).

### 2.3. Obtaining Lipid Vesicles Incorporating DCF

An amount of 0.009 g of L-alpha phosphatidylcholine (egg yolk, Type XVI-E, ≥99% TLC) was dissolved in 1 mL chloroform (≥99.5%); the solvent was removed by evaporation (using a RE-2000A Rotary Evaporator, Ya Rong Biochemical Instrument Factory, City, China); and the lipid film was dried for 4 h at 50 °C using a vacuum pump for 2 h.

**Figure 1:**
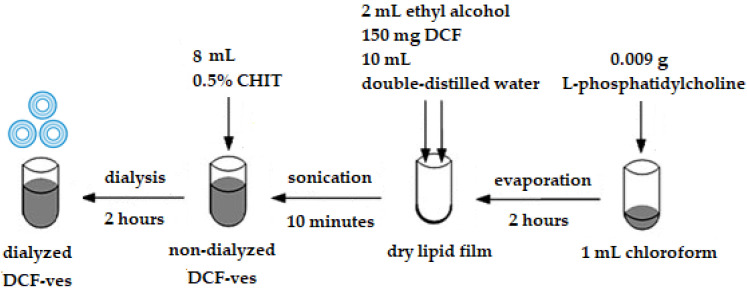
The procedure for obtaining chitosan-stabilized lipid vesicles with DCF.

In 2 mL of ethyl alcohol, 150 mg of diclofenac sodium salt (molecular weight 318.13) were dissolved, and double-distilled water was added until 12 mL, obtaining a 12.5 mg/mL solution at a temperature of 23 ± 2 °C, which was magnetically stirred for 2 h at 800 revolutions/minute. The dry lipid film was hydrated with this solution and then subjected to an ultrasound field with an amplitude of 25% for 25 min/20,000 kJ/25 °C using a Sonoplus Bandelin ultrasound generator (Bandelin electronic GmbH & Co. KG Berlin, Germany) (Figure 1).

The coating of lipid vesicles with chitosan was performed by adding 8 mL of 0.5% chitosan in acetic acid 1% solution over the suspension of the drug-loaded vesicles. The pH of the suspension was adjusted to 4.56 to prevent chitosan agglomeration in a too alkaline environment. The suspension was subjected to magnetic stirring at 800 rpm for 10 min. The vesicles’ suspensions were dialyzed for 2 h in double-distilled water in order to obtain a pH as close as possible to the physiological values. Milli-Q water (18.2 MΩ∙cm) was used to prepare all solutions. Barnstead Easy Pure II purification system was used for water sample deionization. Cellulose dialysis tubes type D6191-25EA, with pores of 12,000 Da MWCO from Sigma Aldrich (Steinheim, Germany), were used for the dialysis of vesicles suspension.

### 2.4. pH Value of Lipid Vesicle Solutions with DCF

The pH of DCF solutions, DCF lipid vesicles and DCF vesicles stabilized with chitosan were determined using the Sartorius Professional PP-50 pH meter (Sartorius Lab Instruments GmbH & Co. KG, Göttingen, Germany).

### 2.5. Optical Microscopy of DCF-ves

Optical microscopy analysis was performed using a Nikon Eclipse Ti inverted optical microscope (Nikon, Tokyo, Japan) using 40× objective and 60× objective (CFI VC Plan Apo Nikon, numerical aperture NA 1.40, WD 0.13 mm) with a total magnification of 400× and respectively of 600×. The differential interference contrast (DIC) examination mode (Leica Microsystems GmbH, Wetzlar, Germany) was used to observe the vesicles’ shape.

### 2.6. Size of Lipid Vesicles with DCF

The dimension of the vesicles entrapping DCF were measured using the Malvern Zetasizer Nano ZS ZEN-3500 apparatus (Worchestershire, UK), and the size distribution was assessed by visual comparison of micrographs, with a standard scale measurement, using a Nikon Eclipse Ti-E inverted microscope equipped with imaging software NIS Elements Basic Research (NIS E-Br).

### 2.7. Zeta Potential of Lipid Vesicles with DCF

The electrophoretic mobility and Zeta potential of DCF-containing vesicles was assessed using the Malvern Zetasizer Nano ZS ZEN-3500 (Worchestershire, UK). In order to analyze the electrophoretic mobility, the samples were diluted with 0.1 mM sodium chloride, after which they were introduced into the measurement cell. Each sample was assessed in triplicate.

### 2.8. The Efficiency Entrapment of DCF in Vesicles

To evaluate the loading degree of DCF in the microsystems, the concentration of the drug in the lipid vesicle suspension was assessed using an ultraviolet visible (UV–vis) spectrophotometer (Hewlett Packard 8453, equipped with a HP Chem-Station software, Waldbronn, Germany). The experiment was repeated three times.

The dialyzed suspension was centrifuged at 5000 revolutions per minute, for 45 min, to separate the vesicles with DCF from the supernatant. The precipitate with DCF-ves obtained after centrifugation was dissolved in chloroform to degrade the lipid vesicles and release the DCF content. The evaporated chloroform was replaced with distilled water up to the initial volume, in the dissolution compartment. The concentration value of DCF released from the lipid vesicles was assessed by comparing the absorbance rate with the calibration curve of the DCF solution. 

The loading efficiency of DCF in the vesicles was determined according to the formula:

Ee (%) = (Wi − We)/Wi × 100;

where Ee is the percentage efficiency (%) of DCF encapsulation; Wi is the initial mass of DCF; and We is the mass of DCF excluded from the vesicles.

### 2.9. Evaluation of Diclofenac Release Kinetics In Vitro

The in vitro release kinetics of DCF from DCF-ves, respectively from DCF solution, was assessed for 10 h using the usual dialysis method. This technique is based on the physical separation of samples, providing the possibility of determining the molar concentration of the drug at repeated time points. Ten mL of the tested solutions were placed in a cellulose acetate dialysis bag (MW 12–14 KDa, Sigma Aldrich, Germany), representing the inner dissolution compartment. The dialysis bag was placed in a large beaker with 200 mL of release medium (consisting of phosphate-buffered saline, pH 7.4, Sigma Aldrich, Germany), which was then subjected to continuous magnetic stirring at 100 rpm at a temperature of 37 ± 0.5 °C.

The molar concentration of DCF was determined using the UV-vis spectroscopy method (Spectrophotometer Hewlett Packard 8453, equipped with HP Chem-Station software for measuring absorbance between 259 nm and 281.6 nm), by extracting 2 mL of medium release at certain time intervals: 15, 30, 45, 60, 90, 120 min, 3 h, 4 h, 6 h, 8 h and 10 h. After each determination, the same volume of dissolution medium was replaced in the assay compartment.

The series of values for each absorbance determination were taken and analyzed using HP ChemStation software (Agilent, Santa Clara, CA, USA). The dissolution profile of DCF released from DCF-ves was compared with that obtained for DCF solution (1 mg/mL), similar to that existing in 10 mL DCF-ves, using the same spectrophotometric method of determination.

The release rate (R) was defined as the number of moles of DCF released per unit time and per unit volume of release medium:

R (t) = 1/V × dν (t)

where V is the volume of the medium and dν is the number of moles of DCF released in the time interval dt.

The molar concentration at a given time (t) was expressed by the relation:

C (t) = ν (t)/V

where C is the molar concentration of DCF released into the environment and ν is the number of moles of DCF at time t in the volume V.

### 2.10. Evaluation of In Vitro Hemocompatibility

The in vitro hemocompatibility study assesses the impact of the test substances on the architecture and viability of erythrocytes by determining the release of hemoglobin into the plasma as a result of erythrocyte lysis after contact with a potentially toxic substance [33,34,35]. Evaluation of the hemolytic properties of lipid vesicles was performed through spectrophotometric quantification of hemoglobin release after exposure to them.

Fresh blood was collected from the lateral vein of the rat’s tail in heparin vacutainers under local anesthesia with 1% benzocaine. The erythrocytes were separated by centrifuging the blood at 5 °C for 5 min at 1500 RCF (relative centrifugal force) and then washed 3 times with saline. As negative control, a 2% (*v/v*) erythrocyte suspension was used, obtained after immersion in saline and incubated for 45 min at 37 °C. Triton X-100 10% (*v/v*), known to possess hemolytic activity [36], incubated for 45 min at 37 °C, was considered a positive control in the experiment. The erythrocyte suspension was incubated with the test substances for 45 min at 37 °C and centrifuged for 10 min at 1000 RCF to remove cells, and the absorbance of the supernatant (including plasma and lysed erythrocytes) was measured at 540 nm using a Hewlett Packard 8453 UV–vis spectrophotometer (Waldbronn, Germany).

The percentage of hemolysis (% hemolysis) was calculated according to the formula [37]:

*% hemolysis = (Test substance absorbance − Negative control absorbance) × 100/(Positive control absorbance − Negative control absorbance)*

### 2.11. In Vivo Biocompatibility Testing of Lipid Vesicles Incorporating DCF

In vivo testing of the study substances biocompatibility was based on the determination of the hematological and biochemical profile of the animals receiving the test substances (bi-distilled water, bladder solution with chitosan, DCF solution, colloidal dispersion of micro-particles with DCF). For in vivo biocompatibility of micro-particles evaluation, 4 groups of 6 animals each will be used, which received the tested substances orally (using an eso-gastric device), according to the following protocol:

Group 1 (Control): 0.3 mL/100 g body weight–distilled water;
Group 2 (CHIT): 0.3 mL/100 g body weight–0.5% chitosan solution;
Group 3 (DCF): 15 mg/kg body weight–DCF;
Group 4 (DCF-ves): lipid vesicles entrapping 15 mg/kg body weight DCF.

At 24 h and 7 days after the administration of the studied substances, the rats were anesthetized with ethyl ether and placed in a plexiglass device for containment. The animal’s tail was immersed in water heated at 40 °C, in order to dilate blood vessels and to collect samples for laboratory examination [38,39]. Local anesthesia with 1% benzocaine solution was performed by placing the animal tail in a rectilinear position, in a previously identified lateral vein, at approximately 3 cm. from the tip of the tail. Subsequently, the area was antisepticized with alcohol (70% *v/v*), and the vein was punctured with a needle tilted at an angle of 20 degrees, for collecting a blood sample of 0.2–0.3 mL [40]. After removing the needle, a gentle pressure was applied to stop the blood from flowing. These blood samples can be used immediately for laboratory tests or kept in the freezer at −80 °C for up to 1 year.

The following tests were done from the collected blood samples: red blood cells (RBC), hemoglobin (Hb), hematocrit (Ht) and leukocyte formula: neutrophilic polymorphonuclear (PMN), lymphocytes (Ly), eosinophils (E), monocytes (M), basophils (B); levels of alanine aminotransferase (ALT), aspartate aminotransferase (AST) and lactate dehydrogenase (LDH); urea, uric acid and creatinine levels; malondialdehyde (MDA), glutathione peroxidase (GPx), superoxide dismutase (SOD); serum complement activity; phagocytosis capacity of peripheral neutrophils (Nitro-Blue Tetrazolium test–NBT test).

Laboratory analyses were performed on special analyzers, separately for each parameter researched. The determination of the hemoleukogram, ALT, AST, LDH, urea and creatinine levels was performed in the PRAXIS Laboratory, Iasi, Romania, with the help of the Hematology Analyzer 5 DIFF model BF-5180 (Manufacturer DIRUI, Istanbul, Turkey).

The serum level of complement was determined using the Hartmann–Brećy technique, based on the hemolysis of sensitized erythrocytes (containing specific antibodies) by serum complement. It is appreciated that a hemolysis of 50% provides much more accurate information about the activity of complement in the blood than total hemolysis [41].

The phagocytosis capacity of PMN in peripheral blood (NBT test) detects metabolic changes that occur during phagocytosis processes, quantitatively correlated with phagocytosis, based on the reduction of "NITRO BLUE TETRAZOLIUM" (NBT–percentage expression) [42].

The Hartmann–Brećy technique was used to determine serum complement activity: serum complement hemolysis sensitized erythrocytes (on which specific antibodies have been attached). The aim is 50% hemolysis, which has a higher accuracy on the activity of the serum complement rather than total hemolysis. For example, if 50% hemolysis is produced by a diluted sample containing 0.1 mL of the tested mixture, the formula: UCH50 = D × 10/0.1 applies.

The determined parameters are part of a battery of tests to evaluate the immunological effects of pharmacologically active agents in experimental research on laboratory animals.

At the end of the experiment, the animals were sacrificed under general anesthesia with isoflurane 5%, and fragments of liver, kidney and stomach were collected, from which preparations were made for the histopathological examination. The harvested tissues were immersed in 10% formaldehyde solution, embedded in paraffin and sectioned at a distance of 5 μm, and Masson trichrome stains were prepared. The prepared blades were further viewed using a Nikon TI Elipse optical microscope, and the images were recorded with a Nikon Coolpix 950 digital camera with a resolution of 1600 × 1200 (1.92 Mpx), optical zoom × 3.

The experimental research protocol was approved, and the Ethics Certificate (No. 24/21.12.2020) was obtained. All the investigations were carried out in compliance with the recommendations of the University of Medicine and Pharmacy Ethics Committee “Grigore T. Popa” Iaşi, Romania, in accordance with national and international standards that concern the protection of animals used for scientific purposes [43].

### 2.12. Statistical Analysis

The data are presented as mean ± standard deviation (S.D.) of mean for 6 animals in a group. The data were centralized and statistically processed using SPSS 19.0 for Windows program and ANOVA method, in order to determine the specific statistical parameters involved in characterization of the distribution series. The differences having the *p*-value less than 0.05 were considered to be significant.

## 3. Results

### 3.1. pH Value of Lipid Vesicle Solutions with DCF

In order for the vesicles to be coated with CHIT, the suspension had to be brought to a pH close to 5, with the addition of acetic acid that serves to prevent polymer agglomeration. The non-dialyzed vesicles incorporating DCF have an acidic pH (pH = 4.56), which is unsuitable for administration to the laboratory animal. For changing the pH to values close to physiological, these vesicles were dialyzed, finally obtaining a colloidal dispersion with a pH of 6.2 (Table 1).

### 3.2. Optical Microscopy of DCF-ves

**Figure 2:**
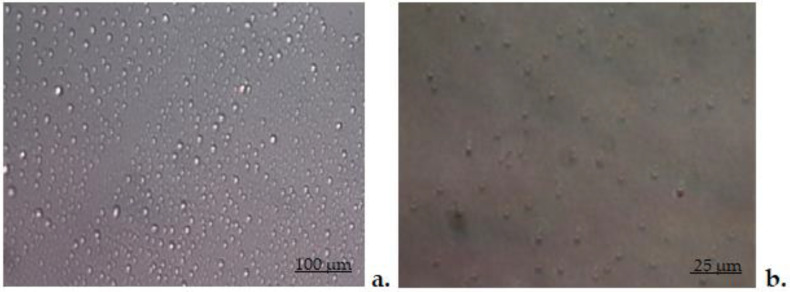
DIC (20×) optical microscopy images of chitosan-free DCF-ves (**a**) and chitosan-stabilized and dialyzed DCF-ves (**b**).

The first image of optical microscopy in Differential Interference Contrast (DIC) shows vesicles with DCF without chitosan. These vesicles show a moderate dispersion throughout the liquid. The second image suggests that, after coating with chitosan, the blisters appear more scattered, but their size increases (Figure 2).

The confirmation of coating of vesicles was given, on the one hand, by the Zeta potential, which showed that the electric charge on the vesicles’ surface changed and became positive, and on the other hand, by the modification in the morphology of the vesicles covered with chitosan, highlighted in the DIC optical microscopy images. The first DIC image obtained at a magnitude of 40× revealed the lipid vesicles loaded with DCF with a non-uniform distribution position, appearing as drops adhering to the surface of the glass support. In the second DIC image, taken with the objective set at 60×, the vesicles coated with chitosan showed a uniform distribution, as strongly hydrophobic drops on the glass support.

### 3.3. Size of Lipid Vesicles with DCF

**Figure 3:**
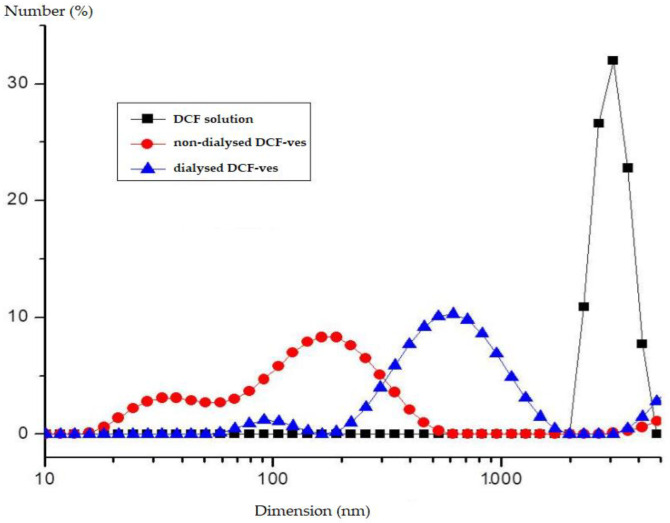
Size distribution of DCF-ves.

The size of DCF-loaded lipid vesicles reached an average value of 102 ± 7.45 nm with a polidispersity index of 0.328. The addition of chitosan increases the size of the vesicles to 562 ± 13.37 nm with a polidispersity index of 0.189 (Figure 3). Due to the fact that the pH of the colloidal dispersion of DCF-loaded lipid vesicles is 7.38, some of the added chitosan crystallized and precipitated. Coating with insufficient layers of chitosan did not allow proper stiffening of the bladder surface.

**Figure 4:**
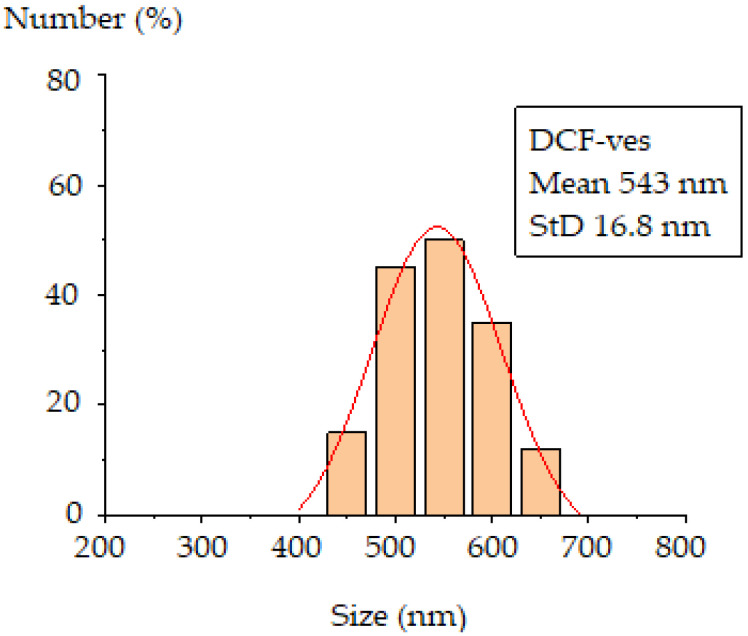
The dimensional histogram of DCF-ves coated with chitosan.

From the dimensional histogram obtained, an average size of 543 ± 10.17 nm results for DCF-ves covered with chitosan, which is in accordance with the hydrodynamic size obtained by DLS assessed with the Malvern device (Figure 4).

### 3.4. Zeta Potential of Lipid Vesicles with DCF

**Figure 5:**
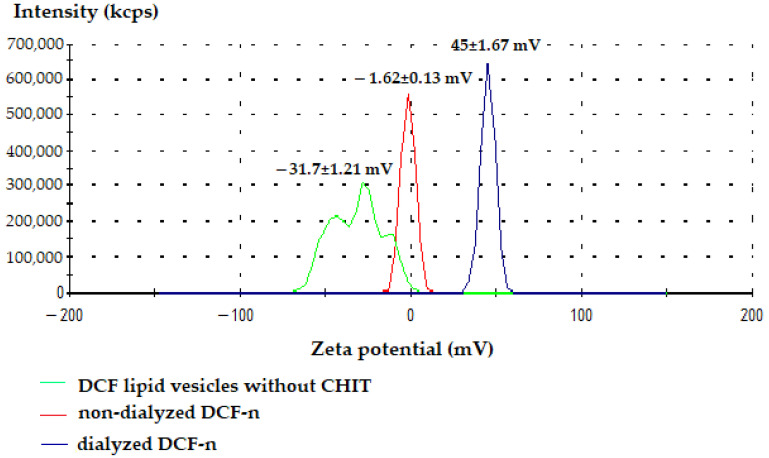
Distribution of Zeta potential for DCF-vesicles.

According to the stability criteria of colloidal dispersions, we can state that the lipid vesicles loaded with DCF showed a moderate stability (objectified by the value of −31.7 ± 1.21 mV of Zeta potential), due to strong rejections that appear between the vesicles loaded with negative charges. The addition of chitosan to the negatively charged vesicle surface caused attraction of positively charged chitosan chains. This led to a Zeta potential of 45 ± 1.67 mV of the colloidal dispersion, an indicator of good stability (Figure 5).

### 3.5. The Efficiency Entrapment of DCF in Vesicles

**Figure 6:**
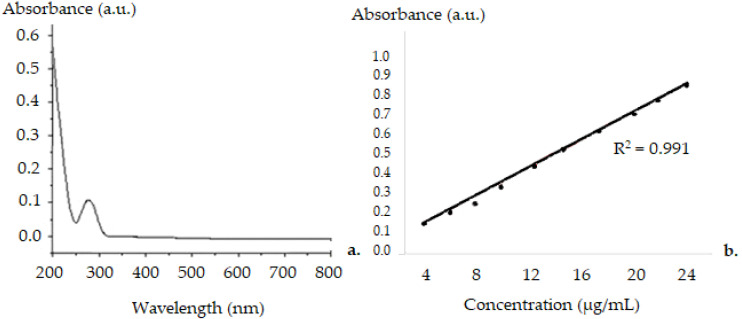
UV–vis Spectra for DCF in suspension (**a**) and calibration curve (**b**).

The calibration curve of DCF in aqueous solution was made (Figure 6) with a mean square deviation (R^2^) of 0.991. The UV-vis spectra of lipid vesicles containing DCF solutions show an absorption peak at 282 nm. The experiment was repeated three times. Considering that the initial mass of DCF was 12.5 mg/mL and that of DCF excluded from vesicles was 2.55 g/mL, the calculated percentage efficiency of drug incorporation into vesicles was 79.4%.

### 3.6. In Vitro DCF Release Profile from DCF-ves

**Figure 7:**
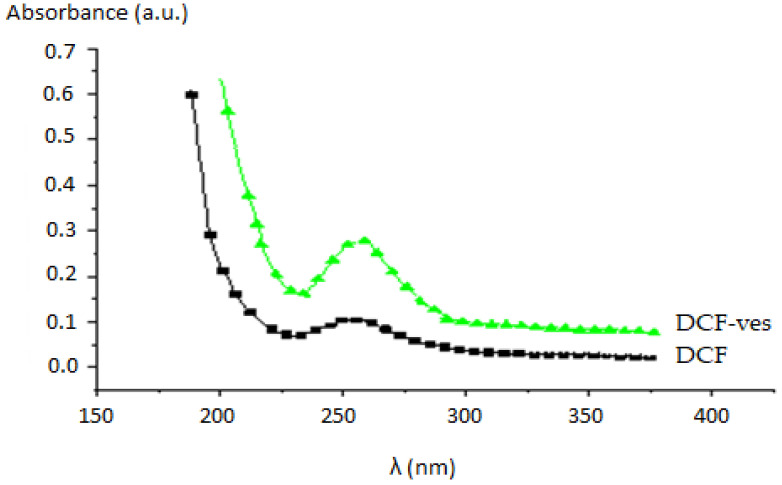
The absorption spectra of DCF and DCF-ves (legend: a.u.–absorption units, λ–wavelength).

The spectrophotometric evaluation of encapsulation degree of the active substance was determined using the calibration curve. To obtain the release kinetics profile, we considered that the average volume remained constant over time (solvent evaporation during the experiment was negligible). The calibration curve of the variation of DCF concentration over time was obtained using the UV-vis absorption spectra (Figure 7).

**Figure 8:**
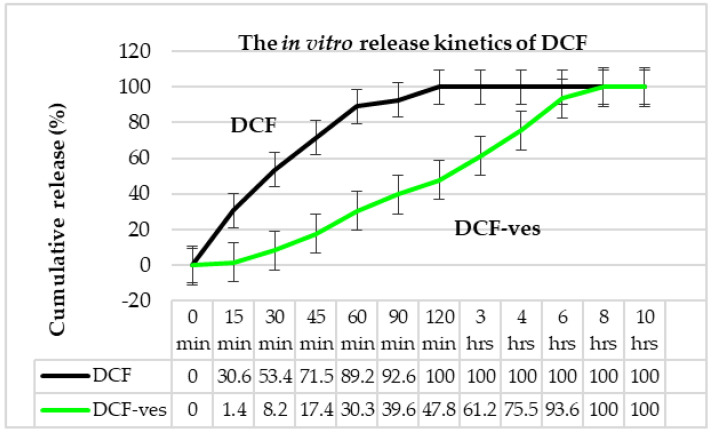
The release kinetics of DCF from DCF-ves, respectively, from the DCF solution by the permeation method.

The measurements were made in the ultraviolet (UV) range, the maximum of the absorption spectra being positioned at 276 nm for DCF, given the fact that neither the solvents nor the other polymeric ingredients used absorb at the given wavelength. In vitro release curve analysis revealed a slower release of DCF from chitosan-stabilized DCF-ves compared to release from DCF solution (Figure 8).

Detailing the aspects of the kinetic profile of the drug, it was found that, after 30 min, 53.4% DCF was released from the DCF solution, while only 8.2% was released from the DCF-ves. Drug release reached a percentage of 92.6% from DCF solution and 39.6% from DCF-ves after 90 min. The active substance was 100% released from the DCF solution after 120 min. Finally, the release of DCF from lipid vesicles appeared to be efficient, reaching a maximum of 93.6% after 6 h (Figure 8). The lower percentage of drug released from DCF-ves was attributed to the high dispersion of the active substance as isolated molecules well-entrapped in chitosan-stabilized vesicles.

### 3.7. In Vitro Hemocompatibility Assessment

The contact of the erythrocyte suspension with Triton X-100 produced considerable hemolysis, with a statistically significant hemolysis rate (** *p* < 0.01) compared to the negative control in the experiment (Table 2).

Incubation of the erythrocyte suspension with DCF was not associated with their obvious hemolysis compared to the negative control group (Table 2).

Exposure of erythrocytes to the test substances (CHIT and DCF-ves) resulted in a low degree of hemolysis, with no significant significance compared to the negative control, thus suggesting good in vitro hemocompatibility.

### 3.8. Evaluation of In Vivo Biocompatibility of Lipid Vesicles with DCF

#### 3.8.1. Hemoleukogram

No statistically significant differences in the number of RBCs were found between the CHIT, DCF, DCF-ves and control groups at 24 h and 7 days (Table 3).

There were no statistically significant variations in serum Hb values in animals treated with CHIT, DCF, DCF-ves compared to animals that received distilled water after one day and one week in the experiment (Table 3).

Substantial changes in Ht values were observed in the animals in which the vesicle solution was administered with CHIT, DCF, DCF-ves compared to the control at the two time points of the determinations (Table 3).

It was found that the levels of PMNs obtained from the analysis of blood collected from rats that received CHIT, DCF and DCF-ves are comparable to those from control animals after 24 h and after 7 days, respectively (Table 4).

No obvious changes were observed with regard to the number of Ly in the peripheral blood between the animals in the CHIT, DCF and DCF-ves groups and those in the distilled water group at the two times of the determinations (Table 4).

No significant changes in the number of M were detected in animals treated with CHIT, DCF and DCF-ves compared to controls during the experiment (Table 4).

Laboratory investigations did not reveal any significant differences in the number E, respectively, the number B between the CHIT, DCF and DCF-ves groups and the control group at the two time points during the experiment (Table 4).

#### 3.8.2. The Activity of Liver Enzymes and Lactate Dehydrogenase

The activity of liver enzymes (ALT, AST), as well as LDH levels in blood, provides information about the liver in conditions of inflammation or functional/structural alterations.

No obvious changes in blood ALT values were observed in animals treated with CHIT, DCF and DCF-ves compared to animals that received bi-distilled water after 24 h and after 7 days (Table 5).

The use of CHIT, DCF and DCF-ves was not accompanied by considerable differences in AST activity compared to the batch with bi-distilled water at the two time points of the determination in the experiment (Table 5).

There were no significant differences in serum LDH levels in the CHIT, DCF and DCF-ves groups compared to the control group at 24 h or 7 days (Table 5).

#### 3.8.3. Serum Urea and Creatinine Levels

The dosage of urea and blood creatinine is important to assess the integrity of kidney function and the ability of the kidneys to eliminate the substances tested.

The administration of the test substances was not associated with significant changes in serum urea levels compared to animals given bi-distilled water at 24 h and 7 days after the start of the experiment (Table 6).

The use of CHIT, DCF and DCF-ves did not cause obvious variations in blood creatinine values compared to the control group at the two time points of the determination (Table 6).

#### 3.8.4. The Activity of Some Markers of the Immune Defense System

No substantial changes in serum complement activity were identified in animals treated with CHIT, DCF, DCF compared to the control group in the experiment at the two time points in the experiment (Table 7).

Treatment with CHIT, DCF and DCF-ves, respectively, was not accompanied by statistically significant changes in the phagocytosis capacity of PMN in peripheral blood, compared to the group that received bi-distilled water after 24 h and 7 days after administration (Table 7).

#### 3.8.5. Evaluation of Some Parameters of Oxidative Stress

There were no significant variations in MDA activity in rats in the CHIT, DCF, DCF groups compared to those in the bi-distilled water group at 24 h and 7 days, respectively (Table 8).

The administration of DCF entrapped or not in the vesicles, respectively, of the vesicle solution with CHIT did not produce significant disturbances of the SOD activity, compared to the control group at the two moments of the determination during the course of the experiment (Table 8).

No statistically significant changes in GPx activity were found in animals receiving CHIT, DCF, DCF compared to the group receiving bi-distilled water in the experiment after 24 h or 7 days in the experiment (Table 8).

#### 3.8.6. Histopathological Liver Examination

**Figure 9:**
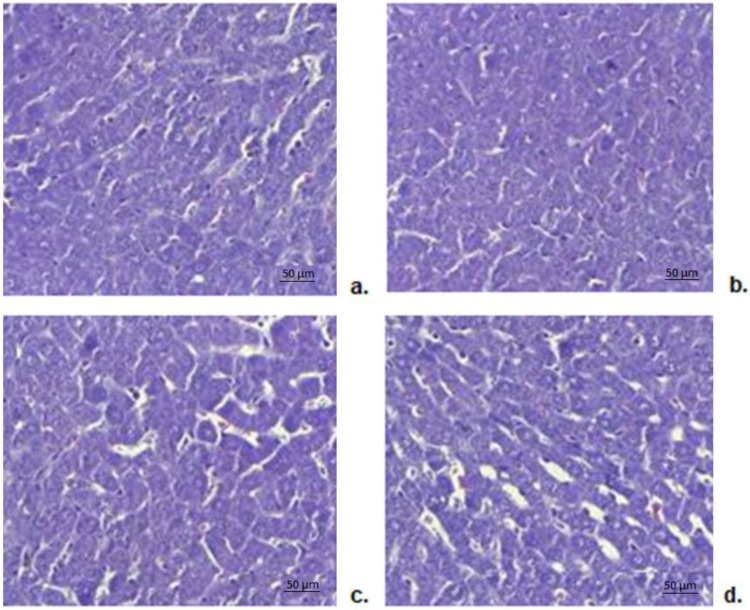
Histopathological examination of liver fragments taken from rats treated with bi-distilled water (**a**), CHIT (**b**), DCF (**c**), DCF-ves (**d**)–Masson trichrome staining × 20.

Optical microscopy analysis of preparations made from liver fragments harvested from animals in the group with bi-distilled water showed a normal-looking morphology (Figure 9a). Hepatic histopathological examination did not reveal any significant alterations in liver structure in animals receiving CHIT compared to the control group (Figure 9b). No obvious liver architecture disturbances were observed in rats in the DCF group compared to those in the control group (Figure 9c). There were no significant changes in liver structure in animals receiving DCF-ves compared to the control group (Figure 9d).

#### 3.8.7. Renal Histopathological Examination

**Figure 10:**
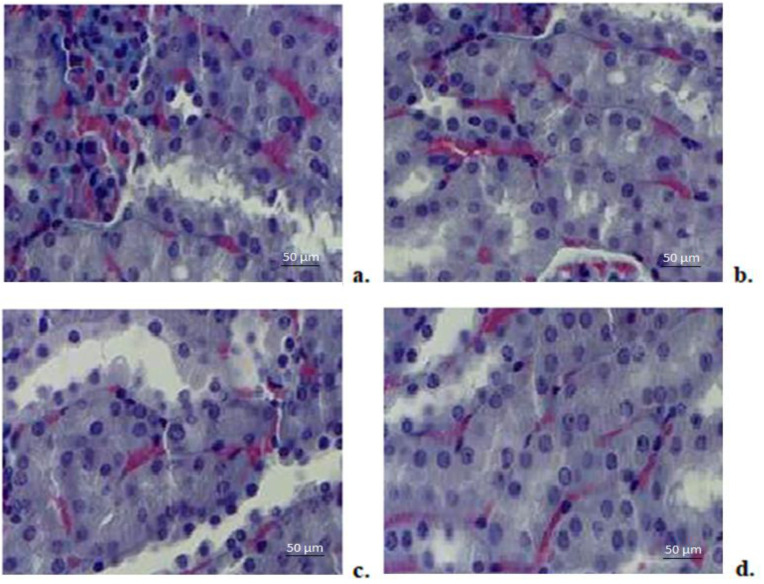
Histopathological examination of kidney fragments taken from rats treated with bi-distilled water (**a**), CHIT (**b**), DCF (**c**), DCF-ves (**d**)–Masson trichrome staining × 20.

Histopathological study of kidney fragments from animals given bi-distilled water revealed a normal architecture (Figure 10a). No substantial disturbances of the renal parenchyma were observed in animals treated with CHIT compared to those that received bi-distilled water (Figure 10b). Administration of DCF to rats did not result in significant alterations in kidney structure compared to those in the control group (Figure 10c). No significant changes in renal architecture were noted in animals using DCF-ves compared to those in the bi-distilled water-treated group (Figure 10d).

#### 3.8.8. Histopathological Examination of the Stomach

**Figure 11:**
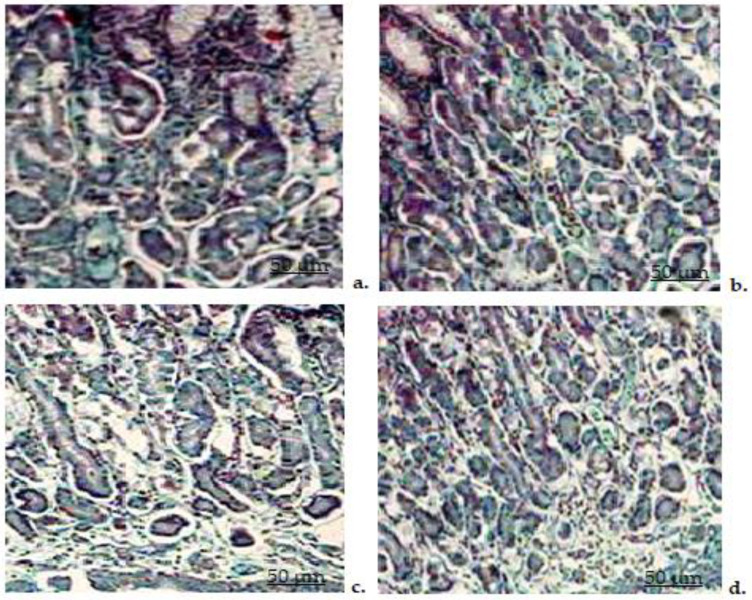
Histopathological examination of stomach fragments taken from rats treated with bi-distilled water (**a**), CHIT (**b**), DCF (**c**), DCF-ves (**d**)–Masson trichrome staining × 20.

Histological examination of stomach fragments taken from animals in the bi-distilled water group revealed a normal structural appearance (Figure 11a). The stomach lining had an intact epithelial surface, muscularis mucosae and normal-looking gastric glands. No significant changes in normal stomach architecture were found in rats receiving CHIT compared to those receiving bi-distilled water (Figure 11b).

DCF treatment was correlated with significant disturbances in the structure of the stomach, caused by the presence of fragmented and disorganized collagen fibers, with rupture of the epithelium on certain parts of the gastric mucosa, blood vessel congestion and infiltration with mononuclear cells in the submucosa, signs of scaling of the gastric glands, which have a vacuolated cytoplasm) (Figure 11c). No significant alterations in stomach architecture were observed in the DCF-ves group compared to the control group (Figure 11d).

## 4. Discussions

Studies have proved that using drug carriers as vesicles with micrometric dimensions decreases drug toxicity [44,45,46].

Various experimental studies have shown that the use of nanoparticles as carriers for NSAIDs has the advantage of significantly reducing renal and gastrointestinal side effects, which are usually produced by the unincorporated drug [19].

DCF is a class II active drug (according to the Biopharmaceutical Classification System) with a high permeability but low solubility [47]. In order to improve the pharmacokinetic profile and the pharmacodynamics effects and to reduce the adverse reactions, especially gastrointestinal, several types of nanoparticles with DCF have been designed, using various materials including: poly lactic-co-glycolic acid; ethylcellulose; chitosan/poly methacrylic acid or poly-pyrol/menthol; polyvinyl alcohol or didodecyldimethylammonium bromide [48,49,50,51,52,53].

Prolonged-release forms of DCF sodium have been developed to improve the safety profile of the drug and to provide a convenient once-daily administration for the treatment of patients with chronic pain [24]. In order to avoid the adverse effects of prolonged-release oral administration, significant efforts have been made over time to prepare nanoparticles that incorporate the drug. Being an amphiphilic substance, DCF tends to form cellulose aggregates, which has led to the design of various formulation techniques to reduce its adverse reactions [54].

Lipid formulations have proved to significantly increase the bioavailability of hydrophobic drugs, such as DCF, compared to conventional dosage forms [55,56]. 

Various studies have shown that the use of DCF sodium microspheres, based on sodium alginate, obtained by ionotropic gelation methods, or by lactide-co-glycolide, improved the release profile of DCF, in vitro but also in vivo [57], while another study demonstrated a high degree of absorption of this anti-inflammatory embedded in sodium alginate/nanocrystalline cellulose/polyvinyl alcohol microspheres functionalized with polyethyleneimine [58].

Other researchers have prepared thin lamellar vesicles of DCF sodium using a thin film moisturizing technique using soy lecithin, cholesterol and ethanol or a mixture of chloroform and methanol as solvents. However, the effects of these vesicles have not been investigated in laboratory animals [59,60].

Goh and colleagues obtained liposomes with DCF by the pro-lipo-Duo method using dimethyl sulfoxide as solvent. Oral administration of these DCF-containing nanoparticles has shown more pronounced anti-inflammatory effect than the unincorporated drug in carrageenan and formalin paw inflammation tests and in the subcutaneous pellet implant granuloma test in rats. It has been suggested that the increased anti-inflammatory activity of DCF nanoparticles may be due to the ability of the liposomal administration system to modify the bio-pharmacological properties of the drug (increasing in solubility, facilitating the absorption through lymphatic transport, prolongation of the gastric transit time) and to exert additional protective effects (preventing unwanted metabolism of the substance and reducing gastric efflux) [61].

Original micro-vesicles entrapping DCF were prepared. The lipid vesicles were covered with chitosan, which led to an increase in their size and to obtaining a much more stable colloidal solution.

Determination of Zeta potential is an important technique for characterizing micro-particle systems to estimate surface load, which may be particularly useful in assessing the degree of physical stability of colloidal dispersions containing vesicles [62].

Zeta potential is a key physical parameter, an important and easy-to-measure indicator that characterizes the behavior of micro-particles in colloidal solutions. The value of Zeta potential indicates the degree of electrostatic repulsion between adjacent particles, similarly charged, in a dispersion [63]. For sufficiently small molecules and particles, a high Zeta potential will provide stability, as the solution will not tend to aggregate. When the Zeta potential is small, the forces of attraction can overcome this repulsion and the dispersion can break and flocculate. It can be seen that colloidal solutions with high Zeta potential (negative or positive) are electrically stabilized, while those with low Zeta potential tend to coagulate or flocculate, depending on the value ranges between them [64].

UV-vis analysis highlighted the fact that soft lipid microvesicles trap, with high efficiency (almost 80%), DCF inside them.

According to the UV-vis spectrophotometry analysis performed 6 months after the synthesis of the vesicles, it was observed that the vesicles remained stable stored in the refrigerator at 4 °C.

The stability of DCF-ves does not seem to depend on the size of the lipid vesicles but rather on the molecular weight of the drug and the chitosan surrounding the vesicle. The study of the in vitro release profile revealed slower kinetics of DCF from chitosan-coated lipid microsystems compared to the release of this drug from DCF solution. The sustained release of this NSAID from DCF-ves can be attributed to the high dispersion of the active substance as isolated molecules well-entrapped in chitosan-stabilized vesicles.

The in vitro investigations showed no hemolytic effects in blood of micro-systems containing DCF. Literature data revealed that usually the in vitro results of the hemocompatibility test are positively correlated with the in vivo hematologic, biochemical and immune profile modifications after the use of the studied substances [65,66].

Following the evaluation of the hematological and biochemical profile, no notable differences were revealed, regarding the investigated biological constants, which makes us appreciate that the administration of the tested substances do not significantly influence the hepatic and renal function. These findings correlate with the absence of structural changes in the liver or kidneys, shown in the histopathological examination.

Moreover, we noticed that the use of non-entrapped DCF was accompanied by significant architectural lesions in the stomach, characteristic for the local irritating action, while the treatment with DCF micro-vesicles does not obviously prejudice the structure of the gastric mucosa. This fact suggests that the entrapping of DCF into soft lipid vesicles stabilized with chitosan reduces the gastric irritant effects of this NSAID in rats.

The phagocytic capacity of peripheral neutrophils and the blood values of complement from rats treated with DCF and DCF-ves were not considerably altered during the experiment, demonstrating that the tested substances do not modify the rat’s immune defense capacity.

Measuring the activity of some enzymes involved in cellular oxidation processes revealed that the treatment with DCF and DCF-ves did not substantially influence the oxidative stress in rats.

## 5. Conclusions

We achieved a novel method for obtaining DCF loaded lipid vesicles which were physicochemical and structurally characterized. Preliminary evaluation using the blood hemolysis assay proved a good in vitro biocompatibility of the DCF-ves.

The use of micro-particles containing DCF was accompanied by similar hematological, biochemical and immune responses with non-entrapped drug and did not significantly influence the oxidative stress in rats. In addition, the histological evaluation did not evidence significant alterations of liver and kidney tissue configuration nor the stomach architecture in rats treated with DCF-ves, compared with control animals. These studies suggest that DCF lipid vesicles stabilized with chitosan may be suitable for in vivo use as drug delivery systems, with future possible medical uses.

## Figures and Tables

**Table 1:** pH values of suspensions with DCF.

Solutions	pH
lipidic vesicles with CHIT	7.55
lipidic vesicles with DCF	7.38
non-dialyzed DCF-ves	4.56
dialyzed DCF-ves	6.20

**Table 2:** In vitro hemocompatibility in CHIT, DCF and DCF-ves. Values were expressed as mean ± S.D. of the mean for 6 animals per group. ** *p* <0.01 statistically significant compared to the negative control sample.

Group	Negative Control	Triton X-100	CHIT	DCF	DCF-Ves
**% hemolysis**	0.02 ± 0.01	85.33 ± 3.26 **	2.26 ± 0.05	2.23 ± 0.03	2.27 ± 0.06

**Table 3:** Serum levels of RBC, Hb and Ht in rats treated with CHIT, DCF and DCF-ves. The data are expressed as arithmetic mean ± S.D. of the average values for 6 animals per group.

Group	Moment of Testing	RBC (mil/μL)	Hb (g/mL)	Ht (%)
**Control**	24 h	6.62 ± 0.84	13.46 ± 1.12	40.86 ± 2.43
	7 days	6.98 ± 0.59	14.13 ± 1.06	40.83 ± 1.80
**CHIT**	24 h	7.34 ± 0.28	14.43 ± 0.24	42.43 ± 1.04
	7 days	6.39 ± 0.56	13.50 ± 1.15	41.56 ± 2.89
**DCF**	24 h	6.59 ± 0.68	13.70 ± 0.77	38.63 ± 4.49
	7 days	5.95 ± 0.06	13.30 ± 0.43	41.56 ± 1.30
**DCF-ves**	24 h	7.03 ± 0.28	13.50 ± 0.70	40.56 ± 2.08
	7 days	6.59 ± 0.28	14.20 ± 0.69	43.20 ± 2.06

**Table 4:** Leukocyte formula in animals treated with CHIT, DCF and DCF-ves. Values are expressed as the arithmetic mean ± S.D. of the average number of elements of the leukocyte formula for 6 animals per group.

Group	Moment of Testing	Leukocyte Formula Elements				
		PMN (×10^3^/μL)	Ly (×10^3^/μL)	M (×10^3^/μL)	E (×10^3^/μL)	B (×10^3^/μL)
**Control**	24 h	2.25 ± 0.55	7.21 ± 1.81	0.47 ± 0.1	0.19 ± 0.1	0.01 ± 0.004
	7 days	1.62 ± 0.49	7.68 ± 0.55	0.37 ± 0.15	0.13 ± 0.21	0.01 ± 0.004
**CHIT**	24 h	1.91 ± 0.41	7.01 ± 0.76	0.51 ± 0.10	0.17 ± 0.08	0.01 ± 0.00
	7 days	1.21 ± 0.07	7.02 ± 0.27	0.31 ± 0.12	0.14 ± 0.13	0.01 ± 0.009
**DCF**	24 h	2.49 ± 1.48	7.16 ± 1.01	0.38 ± 0.01	0.15 ± 0.04	0.01 ± 0.00
	7 days	2.55 ± 0.37	7.65 ± 1.15	0.19 ± 0.07	0.15 ± 0.04	0.006 ± 0.004
**DCF-ves**	24 h	1.91 ± 0.9	7.15 ± 0.97	0.31 ± 0.13	0.13 ± 0.07	0.0033 ± 0.004
	7 days	2.44 ± 1.34	7.25 ± 1.18	0.29 ± 0.15	0.13 ± 0.00	0.01 ± 0.004

**Table 5:** Serum levels of ALT, AST and LDH in CHIT, DCF and DCF-ves groups. Values are expressed as the arithmetic mean ± S.D. of the average values for 6 animals per group.

Group	Moment of Testing	ALT (U/mL)	AST (U/mL)	LDH (U/mL)
**Control**	24 h	54.33 ± 6.18	115.66 ± 11.89	876.33 ± 105.61
	7 days	52.66 ± 5.61	121.00 ± 14.37	893.66 ± 111.58
**CHIT**	24 h	57.00 ± 8.34	119.33 ± 15.55	882.00 ± 110.33
	7 days	56.66 ± 8.58	117.66 ± 17.12	895.00 ± 104.61
**DCF**	24 h	61.33 ± 7.33	116.00 ± 12.61	869.66 ± 102.74
	7 days	56.33 ± 6.74	120.66 ± 11.95	887.33 ± 113.18
**DCF-ves**	24 h	60.00 ± 8.12	119.66 ± 13.34	880.66 ± 109.37
	7 days	58.66 ± 7.28	118.33 ± 14.58	891.33 ± 107.89

**Table 6:** Serum urea and creatinine levels in animals treated with CHIT, DCF and DCF-ves. Values are expressed as the arithmetic mean ± S.D. of the average values for 6 animals per group.

Group	Moment of Testing	Urea (mg/mL)	Creatinine (mg/mL)
**Control**	24 h	35.66 ± 4.28	0.35 ± 0.044
	7 days	42.00 ± 5.47	0.39 ± 0.012
**CHIT**	24 h	43.00 ± 5.58	0.34 ± 0.016
	7 days	38.33 ± 3.54	0.37 ± 0.023
**DCF**	24 h	37.66 ± 5.12	0.37 ± 0.009
	7 days	36.33 ± 4.33	0.41 ± 0.012
**DCF-ves**	24 h	34.66 ± 4.74	0.36 ± 0.032
	7 days	39.00 ± 5.61	0.40 ± 0.009

**Table 7:** Serum complement levels and phagocytic capacity of peripheral neutrophils in CHIT, DCF and DCF-ves groups. Values are expressed as the arithmetic mean ± S.D. of the average values for 6 animals per group.

Group	Complement (UCH50)	NBT (%)
**Control**	52.66 ± 7.47	16.33 ± 2.58
	53.33 ± 8.28	17.33 ± 3.12
**CHIT**	53.00 ± 7.33	16.66 ± 4.12
	53.66 ± 7.74	16.33 ± 4.61
**DCF**	55.33 ± 8.61	17.00 ± 3.74
	54.66 ± 8.12	17.66 ± 4.95
**DCF-ves**	54.66 ± 9.28	16.00 ± 3.47
	54.00 ± 9.33	17.33 ± 3.28

**Table 8:** Serum levels of MDA, SOD and GPx in animals treated with CHIT, DCF and DCF-ves. Values are expressed as the arithmetic mean ± S.D. of the average values for 6 animals per group.

Group	Moment of Testing	MDA (nmol/mg Protein)	SOD (U/mg Protein)	GPx (µm/mg Protein)
**Control**	24 h	24.33 ± 7.33	175.00 ± 14.74	331.33 ± 24.12
	7 days	26.33 ± 9.12	178.33 ± 15.58	339.66 ± 25.47
**CHIT**	24 h	24.00 ± 8.28	174.33 ± 15.33	334.66 ± 23.61
	7 days	25.66 ± 8.74	177.66 ± 17.61	340.00 ± 21.28
**DCF**	24 h	24.33 ± 9.12	175.33 ± 16.28	336.33 ± 26.12
	7 days	26.66 ± 8.33	181.00 ± 15.47	341.00 ± 25.33
**DCF-ves**	24 h	24.66 ± 7.58	176.33 ± 17.12	333.66 ± 23.74
	7 days	24.00 ± 7.61	180.66 ± 14.95	338.33 ± 26.58

## Data Availability

Not applicable.
